# Comparative study between two different staging systems (AJCC TNM VS BALLANTYNE’S) for mucosal melanomas of the Head & Neck

**DOI:** 10.4317/medoral.21132

**Published:** 2016-03-31

**Authors:** Kuauhyama Luna-Ortiz, Madeleine Aguilar-Romero, Verónica Villavicencio-Valencia, Ernesto Zepeda-Castilla, Horacio Vidrio-Morgado, Nathalie Peteuil, Adalberto Mosqueda-Taylor

**Affiliations:** 1 MD. Department of Head and Neck Surgery at the Instituto Nacional de Cancerologia México DF. Department of General Surgery at the Hospital General Manuel Gea Gonzalez; 2 MD. Department of Head and Neck Surgery at the Instituto Nacional de Cancerologia México DF; 3 MD. Department of Surgery at the Hospital 1ro de Octubre, ISSSTE, México DF; 4 Facultad de Psicología, Universidad Nacional Autónoma de México; 5 DDS, MsCd. Departamento de Atención a la Salud, Universidad Autónoma Metropolitana (Xochimilco), México D.F

## Abstract

**Background:**

Mucosal melanoma (MM) of head and neck (H &N) is a rare entity with a quite poor prognosis. Ballantyne’s staging system has been commonly used since 1970. In the 7th edition of the AJCC Staging Manual a new chapter for the staging of TNM Classification system for mucosal melanoma (MM) of the head and neck (H &N) has been introduced to reflect the particularly aggressive biological behavior of this neoplasm. The aim of this study was to analyze and compare among Ballantyne’s staging system vs TNM H &N in terms of overall survival (OS) and disease-free survival (DFS) in a consecutive population of patients with MM in a cancer centre.

**Material and Methods:**

Descriptive analysis of demographic, clinical and pathological variables of MM of the Head & Neck were performed. We compared the survival curves for both systems according to the Kaplan-Meier method using the Log-rank test.

**Results:**

An up-staging migration effect from Ballantyne’s localized disease to moderately and very advanced disease according to AJCC staging system. The 5-year DFS and OS for Ballantyne’s Localized Disease and AJCC Stage III were 31% and 36% vs. 47% and 50%, respectively. For locoregional disease the 5-year DFS / OS were 5% / 10% for Bal-lantyne’s system vs. 13.8% / 17.8% and 0 / 0% for AJCC Stages IVA and IVB, respectively.

**Conclusions:**

In this series, the TNM staging system for MM of the H &N predicted better the prognosis of the disease when comparing with Ballantyne’s system.

**Key words:**Head and neck, mucosal melanoma, AJCC TNM, Ballantynes´s staging system.

## Introduction

Mucosal melanoma is a rare malignant neoplasm that may develop from melanocytes present in the squamous or respiratory mucosa of the head & neck. It represents 0.7 to 1% of all malignant melanomas, 6.7% of melanomas of the head & neck and 3.5% of sino-nasal malignant neoplasms (1). Mucosal melanoma has an estimated 5-year mortality risk of 68% to 89% which is worse than that for melanoma in other body areas (1-3).

There have been near 1000 case reports of mucosal melanomas of the head and neck worldwide in different case series. Most of them were single institution retrospective case series experiences (range 19 to 95 patients) that reported clinical, demographic and pathological characteristics of the disease and included the results of the treatments given to these patients collected through large periods of time (13-44 years); in general, survival is quite poor, even for the early-stage disease and local recurrence and distance metastases are the rule (4-13).

Lesions diagnosed in the paranasal sinuses and nasopharynx are usually large, deeply invading tumours with a significant risk for distant metastases and death (nearly 100%). Even though oral mucosal lesions are diagnosed in less advanced stages, they show a very aggressive behavior and about two thirds of the patients present a local recurrence or distant metastases within 1 year (14,15).

Ballantyne’s staging system had been the most commonly used since 1970; it classifies lesions into three stages depending on the nature of the disease: Local, Regional or Disseminated. However this does not consider the depth of invasion nor the local extension of the lesion (16). In 2004, this staging system was modified by Prasad *et al. * (17) who divided Stage I into 3 subgroups depending on the depth of tumor invasion. However, this subclassification has not gained widespread acceptance and it has not been correlated with prognosis in other centers (11). Staging used by the American Joint Committee on Cancer (AJCC) for cutaneous melanoma is not applicable for mucosal melanoma for several reasons. Unlike cutaneous melanomas, the occult locations in which mucosal melanomas occur preclude sun exposure as a predisposing risk factor. Histologically, primary lesions are characterizaed by nested and single growth of typical melanocytes in the surrounding mucosa, others histopathologic features of mucosa melanoma include frequent angioinvasion and multicentricity. Whereas melanomas arising on skin without chronic sun damage had frequent mutations in BRAF, those mutations were much less common in melanomas occurring on acral skin, mucosal surfaces, and chronically sun-damaged skin. These findings suggest differing routes by which these tumors develop, which may ultimately impact response to treatment. The 7th edition of the AJCC Staging Manual has introduced a new chapter for the staging of mucosal melanoma of the head and neck in order to reflect the particularly aggressive biological behavior of this neoplasm. All lesions limited to the mucosa are considered T3. Advanced mucosal melanomas are classified as T4a (moderately advanced disease) and T4b (very advanced disease). Melanoma in situ is excluded because of the rarity of this entity.

The aim of this study was to describe the salient demographic and clinico- pathological features of a series of MM of the Head & Neck, and compare them in a retrospective manner using the traditional Ballantyne’s staging system (15) and the AJCC staging TNM Classification system in terms of overall survival (OS) and disease-free survival (DFS) in a consecutive population of patients in a cancer referral centre in Mexico City.

## Material and Methods

We conducted a retrospective study that included all the patients with diagnosis of mucosal melanoma of the head and neck admitted in a Cancer Referral Centre at Mexico City from April 1979 to December 2010. This study belongs to the line of research of mucosal melanoma, which has been approved by the Research and Ethics Committee of our institution.

A descriptive analysis of demographic, clinical and pathological variables considered relevant (such as, age, gender, primary site, and nodal and distant metastases) was performed.

Comparisons between both staging systems were done with the following approach: Ballantyne’s Local Disease vs. AJCC Stage III (T3N0M0), Ballantyne’s Regional Disease vs. AJCC Stage IVA (T4aN0M0 and T3-T4aN1M0) and Stage IVB (T4b anyN M0). We also compared the OS and DFS between lesions depending on their primary tumour site (nasal cavity and paranasal sinuses vs. oral cavity).

We computed the survival curves (Disease-free survival [DFS] and overall survival [OS]) for both systems according to the Kaplan-Meier method. In order to compare both staging systems we used the Log-rank statistical test among the groups and it was considered statistically significant a two-tailed alpha value of *P*<0.05. We did not discuss about treatment given to the patients because it was not the primary objective of this work.

## Results

**Figure 1:**
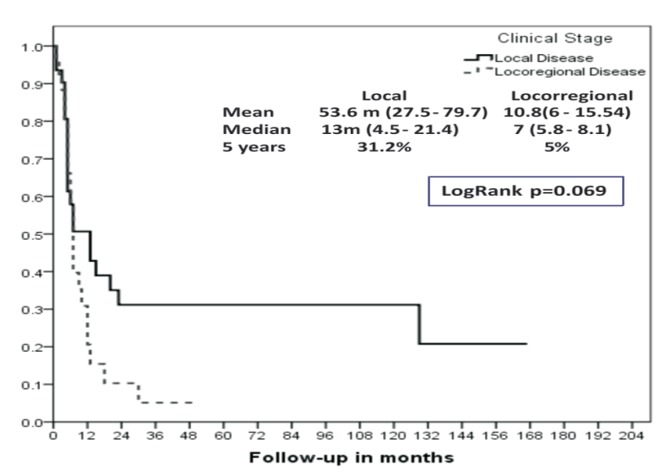
Disease-free survival (DFS) according to Ballantyne’s staging system (Kaplan-Meier).

**Figure 2:**
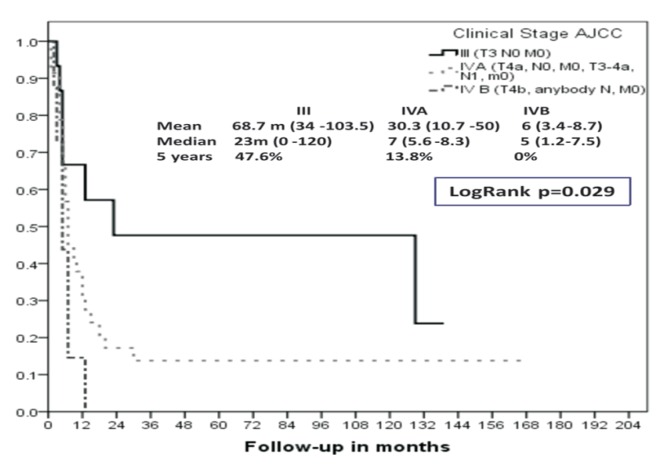
Disease-free survival (DFS) according to AJCC 7th edition staging system (Kaplan-Meier).

We identified sixty-six patients (n=66), 38 females (57.6%) and 28 males (42.4%), with a small predominance of the women (female:male ratio; 1.35:1) and a mean age of 55.39 years (range, 28 to 93 y). Fifty-six patients (84.8%) presented to the hospital because of local symptoms. The mean time between the appearance of the first symptoms and the time of presentation to the hospital was 9.64 months (range 1 to 60 months). Forty-one patients (62.1%) have been previously diagnosed or received any treatment before presenting to this cancer referral centre. Mean size of the primary tumour was 5.1 cm (range, 1 to 11 cm). Demographic and clinical variables as well as comparison between both staging systems (Ballantyne’s vs. AJCC) are shown in  Table 1, demonstrating an upstaging migration effect from Ballantyne’s Local stage to TNM Stage IVA, and at the same time, a reduction in TNM Stage III to 15%. Therefore, there is an increment in Regional disease from Ballantyne’s 42.4% to 66.7% in TNM Stages IVA & IVB (an addition of 24.3%). Metastatic disease remains equal in both classifications. When computing disease-free survival (DFS) for Ballantyne’s Localized Disease we obtained a 5-year rate of 31% in comparison to the 47% observed for AJCC Stage III. In addition, it was calculated for locoregional disease a 5-year DFS of 5% for Ballantyne’s system vs. 13.8 and 0% for AJCC Stages IVA and IVB, respectively (Figs. 1,2).

**Table 1:**
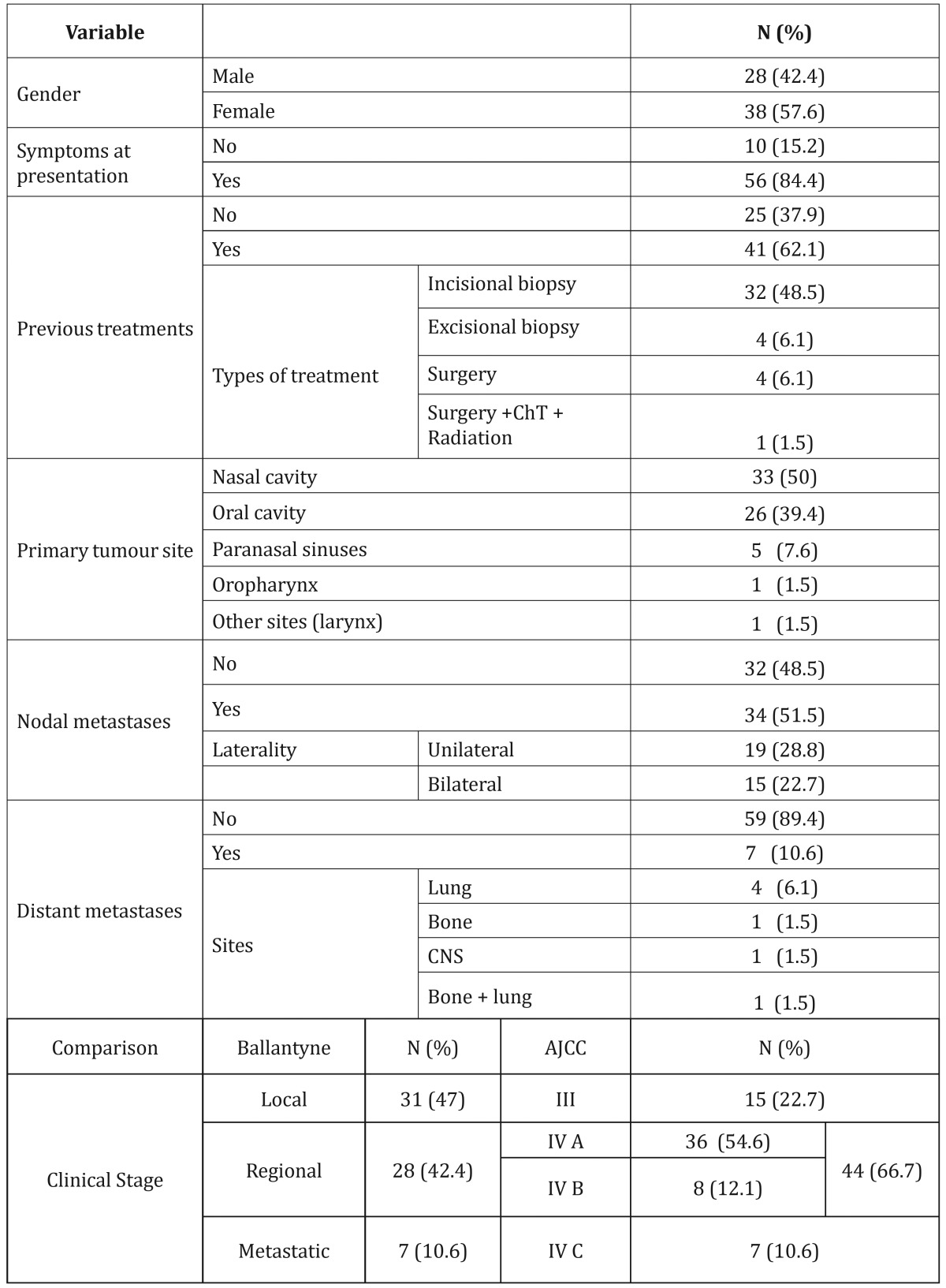
Clinical and demographic characteristics at admission and Comparison between Ballantyne’s and AJCC.

We obtained a 5-year overall survival (OS) for Ballantyne’s Localized disease of 36% vs. 50% observed for AJCC (T3N0M0). The median overall survival for these groups was: 13 months for Localized disease according to Ballantyne’s and 23 months for AJCC Stage III.

**Figure 3:**
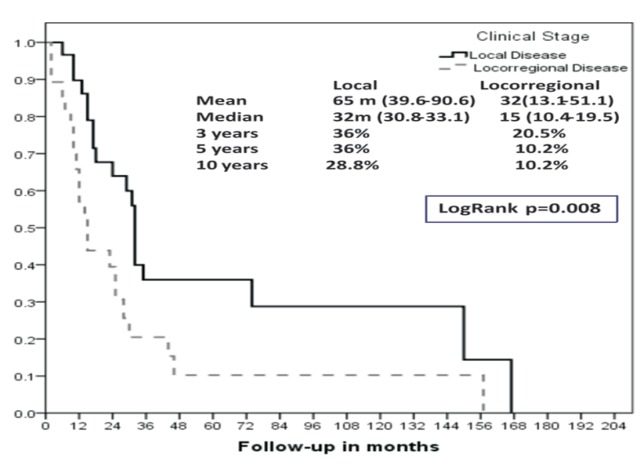
Overall survival (OS) according to Ballantyne’s staging system (Kaplan-Meier).

**Figure 4:**
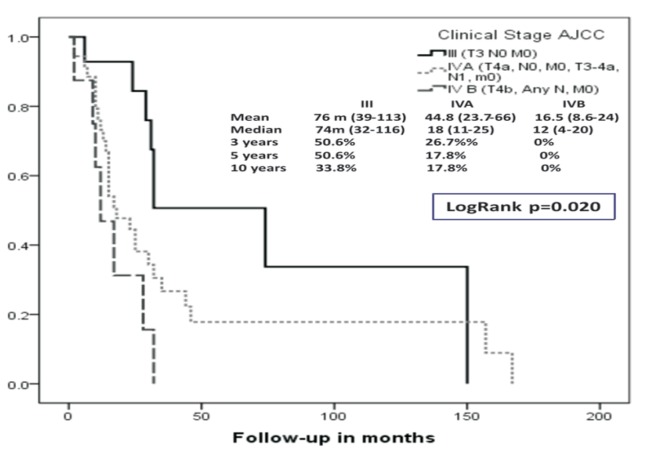
Overall survival (OS) according to AJCC 7th edition staging system (Kaplan-Meier).

There was a 5-year overall survival (OS) for locoregional disease of 10% for Ballantyne’s and 17.8 and 0% for AJCC Stages IVA and IVB (Figs. 3,4). The mean overall survival for these groups was 7 months vs. 7 months and 5 months, respectively.

**Figure 5:**
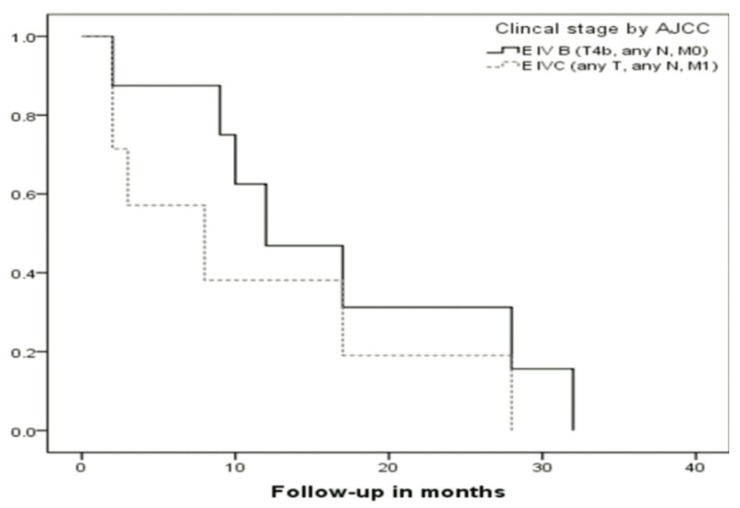
Overall survival (OS) according to AJCC 7th edition staging system (Kaplan-Meier) for AJCC Stages IVB and IVC.

We did not found any difference between OS for AJCC Stages IVB and IVC of the 7th edition of the Staging Manual (Fig. 5). Differences in DFS and OS, observed between groups according to their stage [Ballantyne’s Local vs. Regional and AJCC Stages III vs. IVA vs. IVB] were statistically significant (*P*<0.05)with the exception of the DFS for the Ballantyne’s Local and Regional disease groups.

The comparison between oral cavity vs. nasal cavity plus paranasal sinuses in terms of OS & DFS did not reach a statistical significant difference (*P* = 0.46 & 0.29, respectively).

## Discussion

MM is a rare entity worldwide; despite this, it appears to be more frequent in Japan (18) and it does not seem to have any relation with solar radiation. Ballantyne’s staging was used for decades and it only considered three stages (Local, Regional and Disseminated disease), nevertheless being imperfect, Prasad *et al.* modified Ballantyne’s classification without reaching popularity. In recent years, the AJCC Staging manual in its 7th edition included a different classification system that adequately stages this disease and classifies MM as a minimum as Stage III without Stages I and II, which support the concept of being an advanced disease.

In this study we present a case series that included different mucosal subsites of the upper aero-digestive tract which allowed us to evaluate the utility of the TNM system for MM and to compare it with Ballantyne’s system. The primary tumour site in this series does not differ much with what has been described in other studies, as we found cases in nasal cavity and paranasal sinuses (57%), oral cavity (40%), and other sites (3%). Interestingly, more than one half of the patients presented with nodal disease (51.5 %) and nearly one quarter (22.7%) presented bilateral neck disease, which is quite different with respect to the case series reported in the literature (9.8%) that have focused on the nasal cavity and paranasal sinuses but is accordance to case series with a greater relative proportion of primary tumours in the oral cavity (4-13). In this series metastatic disease at presentation was observed in 10.6%, which is similar to other reports in the literature which ranges from 5% to 10.3% (14,15,17).

Mucosal melanoma presents with a deeper and larger local extension as compared to melanoma presenting in other sites. It has been observed a greater local recurrence rate and a worse prognosis for patients with locally more advanced lesions which motivated the sub classification of the T stage in the AJCC 7th edition staging system and which is not taken into account in Ballantyne’s staging system. It is important to note that in the comparison of the less specific Ballantyne´s system with the AJCC 7th edition system it can be observed a stage migration effect which could explain the observed differences in DSF and OS between both staging systems. (Ballantyne’s Local/Regional disease 47/42.4% vs. AJCC Stages III / IVA + IVB, 22.7/66.7%) ( Table 1).

Of note, even though the AJCC considers that nodal disease is not an important prognostic factor for the mucosal melanoma of the head and neck (in contrast to metastatic disease), any patient with regional disease is considered Clinical Stage IV. In this respect, in this paper we found a statistical significant difference in terms of OS between the patients presenting with and without nodal disease (Figs. 1,3).

We did not find any statistical difference in relation to metastatic disease for both classifications. Similar to the findings of other series, prognosis in terms of DFS and OS was quite poor, especially in relation with recurrence of the disease and later development of distant disease. We did not observe any difference between AJCC 7th edition Stage IVB and IVC in terms of DFS or OS. Both groups of patients in this retrospective series had a very poor prognosis (5-year DFS and OS of 0%). Possibly, this could mean one of two different things, prognosis for Stage IVb should be considered equivalent to that of metastatic disease (Stage IVc) or this study lacked a larger number of patients as to be able to find a difference between these two groups.

In other studies (some based in national registries such as Finland or USA [SEER] it has been compared the classification system for nasal cavity and paranasal sinuses carcinoma (AJCC 6th or 7th edition), which has a greater specific weight given by the primary site of the tumour, to the proposed new system for the mucosal melanoma of the head and neck, which takes into account the greater aggressiveness of this entity and assigns a minimum stage for the primary tumour as T3. In some studies it has been shown that the staging system for mucosal melanoma has a better prognostic performance, meanwhile others have found a better prognostic performance for the paranasal sinuses carcinoma staging system (19-21). So, It has been largely proposed that mucosal melanoma of the nasal cavity and paranasal sinuses has a different clinical behavior and prognosis in comparison with oral cavity, hence, most of the works published in the literature include only a subset of patients with primary tumours arising in nasal cavity and paranasal sinuses. Nevertheless, in the comparison we did in terms of OS and DFS we did not find any statistical significant difference between these subgroups of patients. Both have quite poor prognosis, independently of the primary tumour site. In the work conducted by Michel *et al.* (21), they found a better prognostic performance of the AJCC 7th edition staging system for carcinoma of the nasal cavity and paranasal sinuses applied to the mucosal melanomas arising in these primary sites in comparison with the specific mucosal melanoma staging system for all the head and neck region. In the series here presented, the comparison between oral cavity vs. nasal cavity plus paranasal sinuses in terms of OS & DFS did not reach a statistical significant difference (*P* = 0.46 & 0.29, respectively).

In the case series here presented, the AJCC 7th system for mucosal melanoma of the head and neck predicted the prognosis of the disease with a greater precision in comparison with Ballantyne’s staging system in MM without differentiating the site of presentation. Our results contribute to the controversy, as we did not find any difference in the prognosis and so, we are in favor of a united staging system for all the head and neck region.

Beyond the prognostic factors included in AJCC staging (TNM), several authors have found other clinical and pathological factors (i.e. age, primary tumour subsite [nasal cavity and oral cavity vs. paranasal sinuses and other sites], histologic subtypes [i.e. pseudo-papillar or sarcomatoid subtypes], margins of resection, number of mitosis and ulceration) that have been correlated with prognosis and that could been added to the present classification in order to give an even more precise prognosis (3,12,17).

## Conclusions

In our retrospective series of patients, the AJCC 7th edition staging system for the mucosal melanoma of the head and neck predicted with a greater precision the prognosis of the disease in comparison with Ballantyne’s system. There is an upmigration from local to stage IVA when comparing Ballantyne´s vs TNM, which rise the stage IVA and IVB more than 24%. No differences exist between both classifications for metastatic disease and no difference was found between Stages IVb and IVc in the TNM system. According to our results TNM for MM can be applied to all MM in the head and Neck region.
